# Evaluation of extracts from *Coccoloba mollis* using the *Salmonella*/microsome system and *in vivo* tests

**DOI:** 10.1590/S1415-47572010005000062

**Published:** 2010-09-01

**Authors:** Marcela Stefanini Tsuboy, Juliana Cristina Marcarini, Dalva Trevisan Ferreira, Elisa Raquel Anastácio Ferraz, Farah Maria Drumond Chequer, Danielle Palma de Oliveira, Lúcia Regina Ribeiro, Mário Sérgio Mantovani

**Affiliations:** 1 Departamento de Biologia Geral, Universidade Estadual de Londrina, Londrina, PRBrazil; 2 Departamento de Química, Universidade Estadual de Londrina, Londrina, PRBrazil; 3 Departamento de Análises Clínicas, Toxicológicas e Bromatológicas, Faculdade de Ciências Farmacêuticas de Ribeirão Preto, Universidade de São Paulo, Ribeirão Preto, SPBrazil; 4 Instituto de Biociências, Universidade Estadual Paulista Júlio de Mesquita Filho, Rio Claro, SPBrazil

**Keywords:** medicinal plants, *Coccoloba mollis*, Ames test, *in vivo comet assay*, *in vivo micronucleus assay*, Polygonaceae

## Abstract

The common everyday use of medicinal plants is an ancient, and still very widespread practice, whereby the need for studies on their possible toxicity and mutagenic properties. The species *Coccoloba mollis* has been much used in phytotherapy, mainly in cases involving loss of memory and stress. In order to investigate its genotoxic and mutagenic potential, ethanolic extracts from the leaves and roots underwent *Salmonella*/microsome assaying (TA98 and TA100 strains, with and without exogenous metabolism – S9), besides comet and micronucleus tests *in vivo*.There was no significant increase in the number of revertants/plate of *Salmonella* strains in any of the analyzed root-extract concentrations, although the extract itself was extremely toxic to the *Salmonella* TA98 strain in the tests carried out with S9 (doses varying from 0.005 to 0.5 μg/plate). On the other hand, the leaf-extract induced mutations in the TA98 strain in the absence of S9 in the highest concentration evaluated, although at very low mutagenic potency (0.004 rev/ μg). Furthermore, there was no statistically significant increase in the number of comets and micronuclei, in treatments involving Swiss mice. It was obvious that extracts of *Coccoloba mollis*, under the described experimental conditions, are not mutagenic.

## Introduction

The use of plants for medicinal purposes is one of the oldest forms of medical practice. Besides this practice continuing to be important in primary health care of the less fortunate population in developing countries, the procurement of medications of natural origin in developed countries has increased considerably in the last decades (WHO, 2000). Therefore, special attention should be given to the evaluation of the safety, efficacy and quality of natural products, which includes the evaluation of natural therapeutic agents for genotoxicity/mutagenicity as recommended by national and international regulatory agencies (CNS, 1997; OECD, 2001).

Although there is scientific endorsement of the numerous benefits arising from medicinal plants, their general use should be viewed with caution. Adverse effects can develop due to intrinsic toxicity, adulteration, substitution, contamination, the erroneous identification of plant material, the lack of inspection, and the interaction with other drugs (Rates, 2001; Zhou *et al.*, 2004).

The species *Coccoloba mollis* Casaretto (Polygonaceae) is commonly used for phytotherapeutic purposes under the name of “*Erva da memória”* or the memory herb. The popular use of this plant has been reported as beneficial in cases of memory loss, stress, insomnia, anemia, diminishing eyesight and sexual impotency. The product is manually prepared in extract form with alcohol from the roots and leaves of the plant. Once prepared, it is kept in the dark for about 15 days. Users are recommended to ingest 10 drops of the tincture diluted in water, two or three times a day. The species, popularly known as folha-de-bôlo or falso-novateiro, occurs in shrublands and semideciduous forests in the Parana River basin, in the center-southern region of Brazil (Lorenzi, 2002). Species of the genus *Coccoloba* have been used in astringent form, in the treatment of fever, diarrhea, gonorrhea, hemorrhoids, menstruation problems and uterine hemorrhage (Mors *et al.*, 2000; Cota *et al.*, 2003). Nevertheless to date no information is available in the scientific literature, as to the biological effects on *in vitro* and *in vivo* systems. On considering the genus *Coccoloba*, and the importance of scientific evaluation of medicinal plants of common use|, the aim was to determine the genotoxicity/mutagenicity of leaf and root ethanolic extracts from *Coccoloba mollis* by using *Salmonella*/microsome assaying (Ames test), as well as *in vivo* ± and micronucleus tests.

## Materials and Methods

###  Extracts of *Coccoloba mollis*

A dried, powdered material (from both leaves and roots) was extracted with 95% ethanol at room temperature, the solvent then being removed under vacuum to yield 40 g of root extract (RE) and 85 g of leaf extract (LE). The leaf material underwent successive fractional partitioning by ethyl acetate. These fractions (30 g) were then chromatographed on a silica gel (174 g) column using increasing polarity solvents (*n-*hexane, dichloromethane and ethyl acetate). The root extract also underwent successive fractional partitioning by *n*-hexane and ethyl acetate. The hexane soluble fraction (4,5 g) was chromatographed on a silica gel (163,66 g) column using increasing polarity solvents (*n-*hexane, dichloromethane, ethyl acetate). The major extract compounds, identified by spectroscopy (NMR, ^1^H/^13^C, CG-MS, IV), were a mixture of long chain hydrocarbons, carboxyl esters and 3-taraxerone (a triterpene) in the leaf extract, and two anthraquinones (emodin and physcion) in the root. The plants collected were identified by Ana Odete Santos Vieira, PhD from the Department of Animal and Vegetal Biology of the Universidade Estadual de Londrina, UEL. A voucher specimen was deposited under number Barros, I B 001.

In the *Salmonella*/microsome assay, the extracts were resuspended in dimethylsulfoxide (DMSO), using test concentration limits, as recommended in the literature (Mortelmans and Zeiger, 2000; SBMCTA, 2004). Based on this recommendation, pure substances or extracts should be tested either at a maximal concentration of 5 mg/plate, or up to their respective solubility limit. Thus, the root extract (RE) was tested at a concentration of 5 mg/plate, whereas the leaf extract (LE) was tested at a lower maximal concentration of 3 mg/plate, due to limited solubility. Initially, assaying was carried out with five concentrations based on a logarithmic scale, in order to determine the range of mutagenic concentrations, if present. Based on this preliminary assay, new test concentrations were chosen

Prior to administration to animals, both extracts (LE and RE) were dissolved in DMSO and PBS (phosphate buffer saline), the DMSO concentration not exceeding 10% of the total volume of the mother solution in preparation. They were then filtered with 0.22 μm porosity disposable filters (Millex^®^ - Millipore), and subsequently sterilized. Administration was via gavage.

###  Experimental animals

Fifty-six male Swiss mice (*Mus musculus*), each weighing approximately 30 g, were distributed into control and experimental groups, consisting of seven animals per treatment, eight groups all told. They were kept in the Animal House for Small Mammals of the Department of General Biology, Biological Sciences Center, Londrina State University (CCB/UEL), under controlled conditions as to temperature (22 ± 2 °C), humidity (55 ± 10%) and photoperiod (12 h). The animals were given solid rations and water *ad libitum* during the entire experimental period.

###  Experimental protocols and procedures for *Salmonella*/microsome assaying (Ames test)

The protocol employed was that with pre-incubation, according to Maron and Ames (1983) and Mortelmans and Zeiger (2000). Briefly, 100 μL of culture from each strain (10^9^ cells/mL) were placed in previously sterilized tubes, where upon 100 μL of each extract solution at different concentrations were added, together with 500 μL of 0.2 M phosphate buffer for the assay in the absence of metabolic activation, or the same volume of the S9 mixture with metabolic activation. Tube contents were mixed and incubated at 37 °C for 30 min. 2.0 mL of surface agar were added, the tubes were mixed, and the mixture was poured into a Petri dish with 20 mL of minimum agar. The plates were incubated upside down for 66 h at 37 °C (±0.5). The test was performed in triplicate. For this study, the strains TA98 and TA100 were chosen, which detect the majority of mutagenic compounds, as the first detects frameshift mutations, whereas the second identifies base-pair substitutions (Maron and Ames, 1983).

DMSO was used as negative control. As positive control in the tests without S9, 4-nitroquinoline-1-oxide (4NQO; CAS number 56-57-5), was used at a concentration of 0.5 μg/plate. For the tests carried out in the presence of S9, the positive control was 2-aminoanthracene (2AA; CAS number 613-13-8) at a concentration of 2.5 μg/plate.

The background was carefully examined to check toxicity. Samples were considered toxic when a thinning of the background lawn was observed. This may be accompanied by a decrease in the number of revertants, the absence of a background lawn or presence of pinpoint non-revertant colonies, generally in conjunction with an absence of a background lawn according to Mortelmans and Zeiger (2000). The colonies were counted manually. Samples were considered positive when ANOVA and dose response were both significant when using the Bernstein model (Bernstein *et al.*, 1982; Umbuzeiro and Vargas, 2003). The results were submitted to statistical analysis using Salanal, a program developed by Integrated Laboratory Systems, Research Triangle Park, N.C., USA.

###  Experimental protocols and procedures for *in vivo* comet and micronucleus assays

The experimental animals were distributed into eight groups: (1) negative control; (2) positive control (cyclophosphamide; CAS number 6055-19-2; 50 mg/kg body weight); (3/4) [LE1]/[RE1] (dose 1 of LE and RE - 6.6 x 10^-3^ mg/kg b.w.); (5/6) [LE 2]/[RE 2] (dose 2 of LE and RE - 6.6 x 10^-2^ mg/kg b.w.); and (7/8) [LE 3]/[RE 3] (dose 3 of LE and RE - 6.6 x 10^-1^ mg/kg b.w.).

In group 1 (negative control), PBS was administered via gavage and intraperitoneally, whereas in group 2 (positive control), PBS was via gavage and cyclophosphamide (50 mg/kg) intraperitoneally. In animals pertaining to the groups for determination of extract genotoxicity (3 to 8), LE or RE were ingested and PBS administered intraperitoneally.

Comet testing was according to Tice *et al.* (2000). 20 μL of peripheral blood was withdrawn from each animal by caudal vein puncturing, 24 h after treatment application. This material was carefully mixed with 120 μL of low melting-point agarose (LMP, 0.5%) at 37 °C and deposited on pre-gelatinized slides. The slides were then placed in lysis solution for at least 1 h. After denaturation (20 min) and alkaline electrophoresis (25V, 300mA, 20 min), the slides were neutralized, fixed and kept refrigerated until analysis., when they were stained with ethidium bromide and examined visually using a fluorescence microscope (excitation filter of 420-490 nm and emission filter of 520 nm), according to Kobayashi *et al.* (1995). One hundred cells were analyzed per slide and per animal. The comets were classified as: class 0 - nucleoids with no tail; class 1 - nucleoids with a tail shorter than the diameter of the nucleoid itself; class 2 - nucleoids with a tail 1 to 2 times the diameter of the nucleoid; and class 3 - nucleoids with a tail more than twice the diameter.

In the micronucleus test, a few drops of blood drawn from the caudal vein were placed on slides pre-stained with acridine orange, and immediately cover-slipped (Hayashi *et al.*, 1990). This procedure was performed 48 h after treatment administration (in accordance with the results of positive controls in our laboratory, and coinciding with the highest peak in micronucleus induction). A total of 2000 reticulocytes were examined per slide per animal by fluorescence-microscopy.

The data obtained in *in vivo* experiments were submitted to non-parametric analysis of variance (Kruskall-Wallis), followed by Dunn's test, with α = 0.05, using GraphPad Instat^®^, version 3.02 software.

## Results

In the Ames test, the results indicated that only LE had a mutagenic effect, albeit at very low potency (0.004 rev/μg), whereas under other test conditions, mutagenicity was not observed (Table 1). As to the root extract (RE), mutagenic potency was not noted under any of the conditions tested, although in the TA98 strain in the presence of S9, this extract was highly toxic for *Salmonella* at all the concentrations tested (p~1) (Table 2).

The results obtained in the comet and micronucleus tests suggest that the treatment of animals with the leaf and root extracts from *Coccoloba mollis*, and under the above described experimental conditions, was not genotoxic.

**Figure 1:**
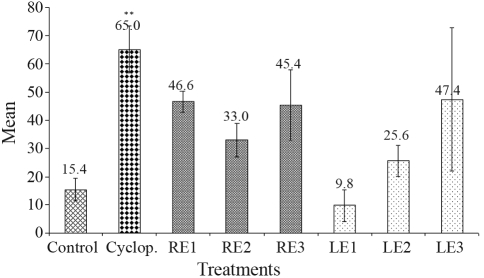
Mean score of comets observed in peripheral blood erythrocytes of male Swiss mice 24 h after treatment with extracts from *C. mollis*. Control: PBS; Cyclop.: cyclophosphamide - 50 mg/kg; RE1: 6.6 x 10^-3^ mg/kg; RE2: 6.6 x 10^-2^ mg/kg; RE3: 6.6 x 10^-1^ mg/kg; LE1: 6.6 x 10^-3^ mg/kg; LE2: 6.6 x 10^-2^ mg/kg; LE3: 6.6 x 10^-1^ mg/kg. RE = root ethanolic extract from *C. mollis;* LE = leaf ethanolic extract from *C. mollis*. **p < 0.01.

The findings of the comet assay are presented in Figure 1. A statistically significant increase in DNA damage only occurred in those animals treated with the DNA damage-inducing agent cyclophosphamide. The same occurred in the determination of micronuclei in reticulocytes. A statistically significant increase in the frequency of micronuclei was seen only in animals treated with cyclophosphamide (Table 3).

A dose-response relationship could be established for the induction of DNA damage in comet assay by LE (r = 0.939), but this was not evident in groups treated with RE with regard to the data on genotoxicty in both assays. In micronucleus test, the animals treated with LE2 and RE2 (6.6 x 10^-2^ mg/kg b.w) showed a higher micronucleus frequency than with LE3 and RE3 (6.6 x 10^-1^ mg/kg b.w), which could be indicative of extract cytotoxicity at this dose.

## Discussion

Interest in phytotherapy is generally motivated by its traditional use and natural origin. Although many beneficial biological activities have been scientifically corroborated, caution is called for in the public use of medicinal plants. Although most phytotherapeutic products are considered safe if used at the recommended doses, untoward effects can occur (Phillipson, 2007). Therefore, genotoxicity/mutagenicity tests, such as those employed in the present study, are important on evaluating safety and efficacy in natural products (Bast *et al.*, 2002). *In vivo* tests and the *Salmonella*/microsome assay are the most frequently used and recommended by regulatory agencies for determining genetic risk (FDA, 1997; OECD, 2001), since they take into account factors regarding *in vivo* metabolism, pharmacokinetics and DNA repair processes (Krishna and Hayashi, 2000) and since the *Salmonella*/microsome assay has a high predictive value for carcinogenicity in rodents when a mutagenic response is obtained (Mortelmans and Zeiger, 2000).

A preliminary analysis of the extracts of *C. mollis* by the *Salmonella*/microsome system shows that they are not mutagenic, except at the highest concentration of the leaf extract (LE) tested in strain TA98, and in the absence of metabolism. Thus, under these conditions this extract possibly induces a frameshift mutation, albeit at a very low level, as this is the type of mutation detected by the TA98 strain. Furthermore, and under the same assaying conditions, it was concluded that toxicity in the root extract (RE) is greater than in the leaf (LE), due the high toxicity displayed in the TA98 strain, in the presence of metabolism. These findings are in accordance with additional data on cytotoxicity from our laboratory, where RE was shown to be more toxic than LE against HTC cells (proficient in drug metabolism) (data not shown). Thus, the biotransformation of compounds present in RE appears to be important in their *in vitro* toxicity. Although comet formation (DNA damage) and the frequency of micronucleated reticulocytes (MNRETs) were increased in animals treated with LE and RE, these were not statistically significant.

The use of *C. mollis* as both a memory modulator and tranquilizer appears to have a certain scientific base. There are reports on the tranquilizing efficacy of plant extracts from the family Polygonaceae (Gosh *et al.*, 2002), besides the protective action of emodin (one of the anthraquinones found in RE) against cerebral disturbances (Gu *et al.*, 2000) and in recuperating memory (Lu *et al.*, 2007), although little is still known about the genotoxic/mutagenic activities of the anthraquinones, especially *in vivo*. Mueller and colleagues have attributed genotoxicity to emodin (Mueller *et al.* 1998,^1999^; Mueller and Stopper 1999), whereas others suggest that it is antimutagenic (Sun *et al.* 2000; Lee *et al.* 2005). Based on studies by Mueller, emodin-induced mutagenicity can be explained by either the formation of 2-hydroxyemodine via CYP1A2 and CYP2B or the inhibition of topoisomerase II, due to the capacity of emodin, with its planar structure, to intercalate DNA. Mutagenicity in the TA1537 strain was shown when investigating the possible mutagenic effects of anthraquinones and their metabolites on different strains of *Salmonella typhimurium* (Tikkanen *et al.*, 1983; Masuda and Ueno 1984; Masuda *et al.*, 1985; Murakami *et al.*, 1987; Westendorf *et al.*, 1990; Krivobok *et al.*, 1992). Although emodin and physcion showed mutagenicity in strain TA1537 of *Salmonella* after metabolism, these anthraquinones were not mutagenic in strains TA100, TA98 and TA2638 either in the absence or presence of metabolism (Krivobok *et al.*, 1992; Mueller *et al.*, 1999; Lee *et al.*, 2005).

Although many mutagenicity studies *in vitro* showed a positive response, a long term study carried out by the *National Toxicological Program* (NTP) of the National Cancer Institute in the U.S. concluded that there was no evidence of emodin possessing carcinogenic activity in male F344/N rats and female B6C3F mice (NTP, 2001). Mengs *et al.* (1997) also did not observe any genotoxic or cytotoxic effects following a single administration of a limited dose of emodin (2 mg/kg b.wt.) in bone-marrow cells of NMRI mice, thus reinforcing the importance of studies in diverse test systems to obtain more reliable conclusions. *In vivo*, the biotransformation of 1,8 dihydroxyanthraquinones (as emodin and physcion) appears to occur in intestinal epithelial and hepatic cells (Mueller *et al.*, 1998), where the biotransformation of emodin is mediated by P450 cytochrome enzymes (Tanaka *et al.*, 1987; Go *et al.*, 2007). Longo *et al.* (2000), on studying the effects of various anthraquinones on metabolic enzymes in the liver and intestine of rats, noted that the 1,8 dihydroxyanthraquinones show the weakest induction of CYP1A2, this induction being significant only in the liver. This observation, together with the possibility that the constituents of the extracts or of their metabolites are free of mutagenic effect or are excreted before causing damage, can explain in part the absence of genotoxicity/mutagenicity effects *in vivo*. On the other hand, it is difficult to state or even suggest a reason for this, mainly as regards LE, since little is still known about the exact composition of this extract. Phytochemical screening using pharmacognostic methodology has revealed the presence of flavonoids and tannins in leaves and roots, whereas results were negative for alkaloids, coumarins, saponins and simple phenolics (Barros *et al.*, 2007, 2008).

Leaf extract from *Coccoloba mollis* analyzed by Oliveira *et al.* (2008), although extracted with different methods, also presented terpenes in its composition (simiarenol and trans-phytol), besides two phytosteroids (sitostenone and sitosterol). Sitosterol, in its beta form, is the most studied of these phytochemicals, and could be involved in the possible cytotoxic effect found in micronucleus test, because it is known to induce apoptosis in many murine and human cancerous cell lines *in vitro*, even at concentrations considered to be very low (Janezic and Rao, 1992; Awad *et al.*, 2000; Moon *et al.*, 2007; Zhao *et al.*, 2009). Studies with oxidised products of β-sitosterol *in vitro* (Maguire *et al.*, 2003; Lea *et al.*, 2004) and *in vivo* (Abramsson-Zetterberg *et al.*, 2007) revealed neither genotoxic nor mutagenic effects.

In conclusion, through the use of *Salmonella*/microsome system and of the *in vivo* assays employed in our work, we can conclude that the leaf and root extracts from *C. mollis* posses low mutagenic response. Further studies are still required for a better understanding of the biological activities of the extracts of *C. mollis*, as they are important for scientifically confirming of the popularly proposed effects and for providing greater safety to people using this medicinal plant. However, the preliminary results, as presented for the first time, can be of aid in guiding such investigations, and contribute to the future registration and validation of this phytotherapeutic medicine.

## Figures and Tables

**Table 1:** Data obtained in the evaluation of the mutagenicity of leaf extract (LE) from *C. mollis* with TA98 and TA100 *Salmonella typhimurium* strains concurrent with or without metabolic activation (S9).

Dose (μg/plate)	TA98 - S9	TA 98+ S9	Dose (μg/plate)	TA100 - S9	TA 100 + S9
0	18 ± 3.00	33.33 ± 6.11	0	139 ± 3.00	102.33 ± 2.89
4NQO (TA98 - S9) or 2AA (TA 98+ S9)	520 ± 105.83*	700 ± 100.00*	4NQO (TA98 - S9) or 2AA (TA 98+ S9)	1030 ± 62.44*	890 ± 85.44
0.3	16.67 ± 1.53	32 ± 2.65	3	140.33 ± 5.03	101.67 ± 6.66
3	17.67 ± 1.53	34 ± 3.61	30	142 ± 9.54	107.33 ± 4.51
30	19 ± 2.00	33.67 ± 4.16	300	138.33 ± 5.86	104.67 ± 8.02
300	23.67 ± 1.15	35.67 ± 2.08	1000	140 ± 11.36	116.33 ± 15.37
3000	31.33 ± 4.16*	41 ± 5.57	2000	148.67 ± 16.26	114.33 ± 9.71

*Significant at 5%. 4NQO (0.5 μg/plate) = positive control in tests without S9. 2AA (2.5 μg/plate) = positive control in tests with S9.

**Table 2:** Data obtained in the evaluation of the mutagenicity of root extract (RE) from *C. mollis* with TA98 and TA100 *Salmonella typhimurium* strains concurrent with or without metabolic activation (S9).

Dose (μg/plate)	TA98- S9	Dose (μg/plate)	TA 98+ S9	Dose (μg/plate)	TA100 - S9	TA 100 + S9
0	24.33 ± 4.51	0	33.33 ± 6.11	0	139 ± 3.00	102.33 ± 2.89
4NQO	520 ± 105.83*	2AA	700 ± 100.00*	4NQO (TA100 - S9) or 2AA (TA100+ S9)	1030 ± 62.44*	890 ± 85.44
5	19 ± 2.65	0.005	T	5	139.33 ± 4.51	108 ± 5.57
50	17.67 ± 2.08	0.05	T	50	142 ± 4.36	116.67 ± 7.09
500	21.67 ± 2.52	0.1	T	100	144 ± 6.0	117 ± 7.0
3000	19.67 ± 2.08	0.25	T	200	140.33 ± 4.51	116 ± 9.85
4000	21.33 ± 1.53	0.5	T	300	151 ± 9.54	114 ± 8.89

T = toxic. *Significant at 5%. 4NQO (0.5 μg/plate) = positive control in tests without S9. 2AA (2.5 μg/plate) = positive control in tests with S9.

**Table 3:** Evaluation of genotoxicity of extracts from *C. mollis*, 48 h after treatment of the animals. Number of experimental animals, total amount of cells analyzed, total number of MNRETs and frequency of micronuclei observed.

Treatment	n	Total of analyzed cells*	Total of cells with MN (MNRETs)	fi MN mean ± SD
control	7	10.000	37	7.4 ± 2.88
cyclophosphamide	7	8.000	132	33.0 ± 5.29**
RE1	7	14.000	66	9.42 ± 3.73
RE2	7	10.000	67	13.4 ± 7.23
RE3	7	14.000	82	11.71 ± 6.1
LE1	7	14.000	82	11.71 ± 3.49
LE2	7	14.000	126	18.0 ± 7.07
LE3	7	14.000	88	12.57 ± 5.85

Cyclophosphamide 50 mg/kg. [LE1]/[RE1] (6.6 x 10^-3^ mg/kg b.w. of LE or RE); [LE 2]/[RE 2] (6.6 x 10^-2^ mg/kg b.w. of LE or RE); [LE 3]/[RE 3] (6.6 x 10^-1^ mg/kg b.w. of LE or RE). * Variable number through the loss of material and not the death of animals during the experiment. **p < 0.01.
